# Copper and Zinc Nutritional Issues for Agricultural Animal Production

**DOI:** 10.1007/s12011-018-1578-5

**Published:** 2019-01-05

**Authors:** Gretchen Myers Hill, Marcia Carlson Shannon

**Affiliations:** 1 Department of Animal Science, Michigan State University, East Lansing, MI USA; 2 Division of Animal Science, University of Missouri, Columbia, MO USA

**Keywords:** Copper, Zinc, Livestock production

## Abstract

Livestock have presented unique requirements and toxicity issues depending on the species for the various concentrations of Cu and Zn and their interactions with other nutrients especially Fe, Se, Mo, and S. Soil concentrations of these elements and their availability to crops influence the health of the crop and the amount found in vegetative tissues and seeds. Hence, many livestock issues are a result of the soils in the area where production is occurring (Loneragan et al. 1981). While water can provide minerals to animals, the amount consumed and availability are highly variable. Many discoveries about Cu were a result of low Cu concentrations and its availability due to interactions with other nutrients in the soils. Anemia, bone disorders, cardiovascular abnormalities, defective wool and hair, and infertility are signs/symptoms of Cu deficiency. Toxicity due to excess Cu is more likely to occur in sheep than other farm species. Swine are tolerant of high concentrations of dietary Cu, and it is often used as a growth stimulant in production. There are many species and physiological stages where the animal’s Cu requirement is not known. Grazing animals can exhibit Zn deficiency when soils and forages contain limited concentrations of Zn. Pastures have been observed to be Zn-deficient in many parts of the world. However, non-ruminant animals usually receive adequate Zn when fed corn and soybean meal diets if there is not excessive Ca and Fe in their diets, but this is not true for rapidly growing young animals. Characteristics of a Zn deficiency include loss of appetite, reduced growth and reproduction, and impaired health of bone and skin tissues.

## Copper

### History

Hart in 1928 first reported that rats needed Cu for growth and hemoglobin formation and then questioned the need for Cu by chicks [2]. Soon the role of Cu in sheep and cattle was recognized [3]. Cattle in Florida lacking Cu were “salt-sick”; sheep and cattle in the Netherlands were “lecksuch,” and Cu deficiency resulted in enzootic neonatal ataxia in Western Australian lambs [4]. Evidence of the importance of Cu when excess Mo was present in the diets of dairy cows was published in 1938 [5].

Because poultry and swine are non-ruminants and consume primarily diets of plant seeds such as corn and soybeans, they are usually not observed to demonstrate Cu and Zn overt deficiencies in production settings where “balanced” diets are fed. However, it has been hypothesized that health might be impaired without overt signs of traditional deficiency [6–8]. The newer molecular laboratory techniques may help to answer this question when applied to production livestock.

### Metabolic Mechanisms and Roles

On a body weight basis, the hepatic content and concentration of Cu are greater in a newborn mammal than at any other time in the life cycle [9]. The Cu, Fe, and Zn concentrations in colostrum are greater than that of later milk in the pig [10]. Mammals provide high hepatic Cu to the fetus and the neonate regardless of the dam’s Cu status [7, 9].

Homeostasis and ultimately protection against toxicity are managed differently in ruminant and non-ruminant animals [11]. Hepatic Cu concentrations increase at lower dietary Cu concentrations such as 5 ppm (sheep) to 10 ppm (cattle) in ruminants, but hepatic Cu is accumulated in pigs until approximately 150 ppm Cu and in hens until 220 ppm Cu. Thus, illustrating that the ability to export Cu via the biliary system and protect against Cu toxicity differs between ruminants and non-ruminants [12]. Using radioactive Cu as oral doses and IV in bred ewes, Moss et al. [13] showed that the amount of Cu deposited in the products of conception increased as the pregnancy progressed. They concluded that a concentration barrier for Cu existed with a syndesmochorial type of placenta found in sheep. Enterohepatic circulation of Cu to maintain Cu status also differs.

#### Sheep

As described by Underwood [14] in his classical text, a Cu deficiency in sheep may present with any of these symptoms: (1) anemia (hypochromic, macrocytic), (2) neonatal ataxia, (3) bone disorders, (4) poor growth and appetite, (5) defective keratinization of wool especially Merinos, and (6) infertility often associated with small, dead fetuses. Neonatal ataxia is also known as “swayback” and “lamkruis” and is a nervous disorder that results in incoordination and, as expected, high mortality.

Since oral supplementation of Cu is more reliable to meet an animal’s needs than depending on the Cu content of plants from the soil, Lassiter and Bell [15] compared oral radioactive forms on blood and plasma Cu concentrations. They reported that the chloride form produced the greatest increases, and the oxide needles and powder were the poorest absorbed forms. In cattle, trace mineral injections (Zn, Mn, Se, Cu) had no benefit in reproducing dairy cattle [16].

Littledike and Young [17] utilized crossbred wethers to determine breed susceptibility to hepatic Cu loading. Lambs sired by Finn, Romanov, and Montadale rams were lowest, Dorset sired were intermediate, and Texel sired had highest hepatic Cu concentration. Woollims et al. [18] utilized three generations of crossbred Scottish Blackface and Welsh mountain lambs selected for low or high plasma Cu concentrations and found that the low Cu lambs had higher mortality rates at all ages and that “Cu deficiency enhanced the susceptibility to infection.” It has been hypothesized that these breed differences may be survival adaptations that occurred due to the locale where the breed evolved.

Using Dorper (meat breed) and Hu (wool breed) crossbred lambs in a comparative slaughter study, Zhang et al. [19] reported that the Cu requirements during growth decreased from 2.86 to 2.18 mg/kg of empty body weight for females and 3.45 to 2.82 for males. Underwood and Suttle [12] note that in estimating the Cu requirement of sheep, besides age and physiological state, consideration must be given to the amount of Fe, S, Mo, and Zn in the diet that affect Cu absorbability. Hence, the requirement on a dry matter basis varies from 4.3 to 28.4 ppm.

Some breeds are more susceptible to Cu deficiency and others to toxicity such as Texel and dairy breeds. Low intakes of Mo increase the risk of this toxicity as does the fertilization of sheep pastures with poultry manure and swine manure [20] from nursery pigs fed pharmacological Cu. Goats are less susceptible than sheep to large intakes of Cu estimated to be about 80 ppm Cu [21]. Of interest, lambs fed once daily accumulate higher concentrations of Cu than when they are fed every 4 h [22].

Internal parasite management especially *Haemonchus contortus* (barber pole worm) is a major problem in sheep production. This is particularly a problem as sheep and goats have developed resistance to currently available anthelmintics (dewormers). Among the management tools utilized today, Cu oxide wire particles may be useful in reducing parasite load and reducing contamination of pastures in sheep and goats [23–26]. Procedures and care for using this technology have been described by Hale et al. [21].

Mehra and Brenner [27] found a form of metallothionein (MT) in pigs fed high concentrations of dietary Cu that was not present in Cu-loaded sheep yielding one of the first differences in Cu handling mechanisms between Cu sensitive and non-sensitive species. Ivan [28] reported that naturally fauna-free sheep had higher hepatic Cu concentrations suggesting that ciliate protozoa increased the sulfide in the rumen that became bound to Cu reducing its absorption and concentration in the liver. Hence, interaction with S and gut physiology also influences the ability of sheep to “tolerate” Cu. Parasites in the gastrointestinal tract have been observed to decrease Cu status and may lead to signs of a Cu deficiency such as swayback of which goats and sheep are susceptible [29]. Thus, suggesting that parasitism and Cu deficiency are associated in many countries that suffer from a “dry” season. The concentration of Cu in pasture is lowest in the winter, and the addition of lime, to increase soil pH from acidic to close to neutral, increases Mo in plants that can reduce Cu availability to 30 to 60%. The Cu/Mo ratio should be greater than 4.5:1 [30].

The toxicity due to Cu depends on the route, source, and quantity of the intoxicant. The target organs in an acute poisoning due to ingestion are gastrointestinal irritation, necrosis, intravascular hemolysis [31, 32], liver failure, [33], and shock, while injection results in liver and renal failure. When the liver’s capacity to manage Cu is exceeded, a chronic Cu poisoning presents as an acute hemolytic crisis with the release of free-unbound Cu in the bloodstream. As noted by Duncan [34] in an acute Cu toxicity, an animal is observed to have abdominal pain with vomiting and diarrhea. Sudden death usually is seen with chronic poisoning.

It is well known that Cu, Mo, and S interact in ruminant animals. The use of intravenous ammonium tetrathiomolybdate has been successful in treating Cu toxicity by preventing the hemolytic crisis and reducing tissue damage [35]. The red blood cell lysing usually occurs under stress when liver cell membranes are destroyed. If given orally, it prevents the accumulation of excessive hepatic Cu. The Cu concentrations in the liver were decreased, but concentrations were increased in the kidney by giving oral tetrathiomolybdate. The lambs from ewes that had increased hepatic Mo concentrations had reduced hepatic Cu concentrations [36]. In this three-way interaction [14], Mo and hepatic Cu are redistributed to many organs [37].

Zinc’s interaction with Cu [38] has been used to treat Wilson’s disease patients who suffer from the metabolic mismanagement of Cu [39] and reduce the incidence of Cu toxicity in sheep [40].

#### Cattle

In the world, Cu deficiency is one of the most common problems in cattle with clinical and subclinical signs. It is most likely to occur when Mo is excessive in the soil and causes severe diarrhea. The availability of Mo in soil and hence the plants is related to pH of the soil.

Puberty is delayed and fertility is depressed, immune system is depressed, hair color may be altered, as well as rough, lameness with bone fractures, anemia, and sudden death due to ruptured blood vessels or myocardial atrophy may occur in Cu deficient animals. Even marginal Cu deficiency (6 to 7 ppm dietary Cu) depresses blood neutrophil function in dairy cattle [41].

Reduction of hepatic and serum Cu concentrations have traditionally been used in examine Cu status [42, 43], but these may not always be appropriate. Usefulness of hair analysis is affected by the differing analytical preparation methods, species, location of selected sample on the shaft, etc. [9] but may be useful in evaluating long-term changes in status. Additionally, cattle breeds differ in their management and need for Cu [44–49].

House and Bell [50] validated the Cu requirements of the dry dairy cow by collecting the products of conception from190 to 270 days of gestation. They reported that Cu accumulated in the conceptus at a rate of 1.6 mg/day.

Of interest is the relationship of Cu with lipids. In 2000, Engle et al. [51]. reported that 20 ppm of Cu could reduce back fat, serum cholesterol, and polyunsaturated fats in steers fed a high concentrate diet. However, in Simmental steers, Cu supplementation had no effect [52]. These researchers did not find any effect on the expression of several lipogenic genes [53].

In many locations in the United States, Argentina, and Canada, forages and grains have been reported to be marginal or deficient in Cu [54, 55]. Primigravid Holstein heifers have been shown to become Cu deficient if Cu supplementation is not provided. An injectable trace mineral supplement (Zn, Mn, Se, Cu) was not shown to improve dairy females’ first conception rate in an intensively managed herd. Blood parameters may not be adequate to detect Cu deficiency. Neutrophil function may be affected [41], and cattle may not be able to respond to intracellular pathogens [56]. When Cu intake is inadequate, blood concentrations may appear normal because of the mobilization of Cu from the liver [57], but metabolic functions are affected [58]. A marked depletion of cytochrome oxidase in gastrointestinal tissue has been observed in Cu-deficient animals and is thought to be related to the diarrhea observed in deficient cattle [59, 60]. An increase in DNA damage has been reported in Cu-deficient cattle that may be related to inability of the animal to provide antioxidant protection.

In Holstein males with an induced Cu deficiency, blood and liver concentrations and ceruloplasmin level decreased by 100 days and changes in hair color, diarrhea, lesions in connective tissue, and cardiovascular lesions after 167 days [60]. More recently, Legleiter and Spears [61] have shown that plasma diamine oxidase is functional in determining Cu deficiency especially when interactions with other nutrients are present.

#### Swine

Pigs, a non-ruminant animal, seldom need supplementation to meet their minimum needs if feed grains are the basis of their balanced diet. Braude (1948) noted that pigs that “licked” Cu pipes grew faster and later reported growth enhancement with pharmacological concentrations of 125 to 250 ppm Cu [62]. Toxicity is observed at 500 ppm ([63, 64] and 1000 ppm results in a more sudden lethality [65].

Estimates of the Cu requirement for swine of different ages and physiological stages have been made, but there is no research for their basis [66]. When semi-purified diets were fed to pigs, Myers [6] estimated the young growing pig required 10 ppm Cu to grow and have adequate hemoglobin. When sows were fed 5000 ppm Zn in corn and soybean meal diets, neonatal pigs lacked adequate Cu in their liver and served as Cu-deficient pigs in research trials to estimate their requirement at 5 to 10 ppm Cu [67].

Of practical importance is the interaction between Fe and Zn. Older facilities with excessive rust (Fe oxide) and high Fe contamination from many feedstuffs result in high concentrations of Fe in the diet that is not available for absorption but interact with Cu in the gut and reduce Cu absorption. Long-term feeding of pharmacological Zn (2000 to 3000 ppm Zn oxide) to nursery pigs and occasionally in finisher diets will result in the depletion of Cu [68]. Metallothionein is a protein produced in response to pharmacological Zn, and it preferentially binds Cu making it unavailable for absorption; hence inducing a Cu deficiency [68].

Pharmacological Cu (125 to 250 ppm Cu) in swine diets improves feed efficiency and growth in nursery, growing and finishing pigs. However, its mode of action is not clear. Research has suggested improvement of gut morphology and microbiota, but no mechanism has been proven. Maribo [69] noted that there was no difference in performance or Cu concentration in the feces when Cu was provided as sulfate or lysine which is similar to the results of Coffey et al. [70] and Veum et al. [71] when sulfate and proteinate Cu sources were compared.

Creech et al. [72] reported that if 15 ppm Cu was fed vs. 25 ppm during the nursery phase, fecal Cu was reduced but performance was not. Exploring the impact of Cu sources on the small intestine, Fry et al. [73] found that when Cu sulfate was compared to tribasic Cu chloride (TBCC) in nursery pigs, there were fewer indications of oxidative stress with TBCC [74].

#### Horses

Few studies have been conducted to determine trace mineral requirements in horses and ponies. Utilizing isotopes, Cymbaluk et al. [75] found that bile was the primary route of endogenous Cu excretion and estimated the dietary Cu requirement for maintaining ponies to be 3.5 ppm.

#### Poultry

Hart et al. [2] ask the question about poultry needs for Fe and Cu soon after they were found to be required by rats. The metabolic role of Cu in poultry has been investigated once requirements were estimated both with and without stress [76]. Lilburn and Leach [77] investigated if abnormal cartilage cells associated with tibial dyschondroplasia were similar to the cartilage of Cu-deficient birds, but a conclusion could not be made. Applegate and colleagues [78, 79] studied the influence of Cu on microbiota, pH, and size of the organs of the digestive tract. Koutsos and Arias [80] reported that the addition of Cu sulfate or TBBC fed to broilers resulted in better performance and fewer intestinal lymphocytes than with Bacitracin and Roxarsone supplementation. Bioavailability of different Cu sources using different end points has been investigated by many researchers [81, 82].

Of importance is the concern relative to oxidant influence of Cu. Luo et al. [83] looked at the effect of Cu sources on vitamin E stability; Miles et al. [84] found that size of particle utilized influenced the amount of oxidation measured. The enzyme Cu Zn superoxide dismutase is part of the biological system essential in reducing oxidative stress and has an important role in preventing aortic rupture in chicks [85] as does lysyl oxidase. Because lysyl oxidase requires Cu for activity, activity is reduced when a Cu-deficient diet is fed [86].

The use of phytase sources affects many nutrients including the interrelationships with Ca, P, Zn, and Cu [87], and many have continued this type of research. Feeding 250 ppm Cu affects P retention regardless of Cu source [88]. Concern relative to Cu toxicity in chicks has led to looking for mechanisms that might be involved [89]. This may be especially relevant since pulse feeding high concentrations of Cu has been reported to reduce cholesterol in breast tissue [90].

Because of concerns about feet and legs in birds, not only has research investigated numerous nutrients in the feed of the animals but the effect of hen dietary components on chick embryos [91]. Kim et al. [92] found that the Cu concentration of egg yolk and feathers could be used to determine relative bioavailability of Cu to hens.

Utilizing several statistical techniques and parameters for hen health and egg production and quality, Berwanger et al. [93] have estimated the Cu requirement of broiler breeder hens to be 12.5 ppm. Their study utilized Cobb 500 hens that were fed 4 weeks of a Cu-deficient diet prior to the study.

There is limited Cu research with turkeys except relative to feet and leg health [94]. Interactions with other nutrients such as Mo are a concern [95].

### Interrelationship of Cu with Other Minerals

Besides the important interaction of Cu with Mo and S in ruminants previously described in sheep, Mo and S can independently affect Cu. Cattle grazing tall fescue (*Festuca arundinacea* Schreb) have been reported to have reduced hepatic and plasma Cu concentrations and ceruloplasmin ferroxidase activity [96] that could be restored by Cu supplementation with this type of pasture forage [97]. Fly ash is known to contain high amounts of Mo, S, and sulfate and has been shown to induce Cu deficiency in cattle, sheep, and horses [98].

The interrelationship with Fe was reported in Cu-deficient pigs fed a low Cu diet where hepatic Fe was twice as high compared to nursery pigs fed 5 to 10 ppm Cu with 50 times higher hepatic Cu [67]. However, when Fe was supplemented (0 to 150 ppm) in nursery pig diets, there was no effect on hepatic Cu, but hepatic Fe was four times greater as supplementation increased [99]. When pigs were fed 250 ppm Cu from weaning to slaughter, pigs developed severe anemia with high Cu concentrations in the liver and decreased Fe concentrations in the plasma and liver [100]. When mature cows were injected (subcutaneous) with 480 mg Cu in disodium EDTA or glycinate, plasma Cu peaked after 2 h and severe hepatic necrosis was found. Plasma Fe increased in 2 to 4 h and remained elevated for 8 h. No glycinate injected cows died [101]. Similarly, subcutaneous injections of 120 mg Cu from disodium edetate resulted in massive liver necrosis and high mortality.

Iron is known to increase in fall forages, and Cu absorption is reduced. However, Underwood and Suttle [102] hypothesized that the Fe × Cu interaction where Cu absorption is affected in ruminants may be a result of the S in the diet not just Fe. This was supported because a Fe × Cu interaction has not been seen in preruminant calves or in sheep fed a low concentrate diet low in S.

While Al has not been reported to alter Cu concentrations, a disorder called Simmental multifocal symmetrical encephalopathy has been reported in Canada, Australia, and New Zealand. In case studies, Al concentrations were elevated and Cu and Mn concentrations were deficient in the liver. These three nutrients are not known to interact, and the observations may be related to breed.

## Zinc

### History

After the discovery of Zn as a required nutrient in rats and mice, scientists found that farm animals required Zn in the 1930s and 1940s. The classic research of Tucker and Salmon [103], who reported that high Ca diets fed in swine production resulted in parakeratosis and could be cured by adding more Zn to the diets, stimulated others to study the Zn metabolism and physiology in farm animals.

Underwood [14] noted in his classic review that there are many Zn-deficient soils in the world that result in pastures and crops that are low in Zn. Hence, clinical signs of marginal Zn deficiency such as reduced growth and appetite, lesions of the skin and its outgrowths of hair, wool, and feathers, and poor reproduction exist in production agriculture. This is especially a problem when antagonists such as phytate found in plant proteins and excessive Ca and Fe are found in the diet.

As an element, Zn is relatively non-toxic and vomiting usually occurs after a high dose of Zn is ingested. Symptoms will depend on the Zn source and length of time and may include dehydration, electrolyte imbalance, abdominal pain, nausea, lethargy, dizziness, and muscular incoordination [104].

### Metabolic Mechanisms and Roles

Zinc can be found throughout the body and serves as a component of many enzymes from those involved in transcription, intra- and intercellular signals to the cell transcription machinery, protein carriers to binding of amino acids to maintain structure, etc. In enzymes, Zn is involved in structure as well as managing valance for the enzyme’s activity and is known to be essential in vitamin A transport. Zinc stimulates the production of MT that serves as storage and detoxification sites and plays a role in Cu/Zn interactions.

#### Sheep

Zinc has many roles in immunity and disease resistance. When lambs were fed a Zn-deficient semi-purified diet, Droke and Spears [105] reported that there was reduced blastogenic response to a T cell mitogen and an increased response to a T-dependent B cell mitogen. Lambs had a lower percent of lymphocytes and a higher percent of neutrophils.

As previously noted, sheep are exceedingly susceptible to Cu toxicity. When Zn was supplemented to sheep, three fractions of Cu and Zn-containing hepatic proteins were found. The 10,000 molecular weight MT fraction had increased Cu binding when the sheep’s diets increased from 2.2 to 11.3 ppm Cu but did not increase when the diet contained 47 ppm Cu. In the intestinal mucosa, there was no Cu and limited Zn associated with this same MT fraction. Certainly, the limited ability of sheep to synthesize MT may be related to the depressed Cu handling by sheep [106].

Beeson’s laboratory [107] was the first to establish the Zn requirement for sheep using semi-purified diets. Of interest with limited validation, sheep appear to require twice as much dietary Zn for quality wool production, male fertility, and maximum plasma Zn than for growth (32 vs. 17 ppm Zn).

#### Cattle

As expected, cattle require Zn for adequate performance, but death occurred only when calves were fed excessive Zn (approximately 1.5 to 2.0 g/day) with a cumulate dose of 30 to 66 g Zn [108].

Cow/calf pairs on pasture benefited when they were supplemented with Zn resulting in increased weight gain [109]. However, levels of Cu, Co, Mn, and Zn fed in combination above recommended amounts reduced reproductive performance [110]. When over 700 ppm Zn was fed to preruminant calves, performance was reduced [111]. Calves fed 706 ppm Zn had an increase in segmented neutrophils and a decrease in eosinophils, prothrombin time, and activated partial thromboplastin time [112].

Wright and Spears [113] found that Holstein steers had similar performance to Zn sulfate and proteinate when 20 ppm Zn was fed. However, when 500 ppm diets were fed from these two sources, Zn concentrations were higher in the duodenal, hepatic, and renal tissue with the proteinate source. Hoof wall contained three times more Zn than the hoof sole, and wall samples from steers fed sulfate were higher than proteinate. When crossbred beef steers were fed these two Zn sources and an unsupplemented control, Zn fed steers had higher performance, quality grade, yield grade, marbling, and backfat regardless of source than control steers [114].

Increasing supplemental Zn in the diets of lactating dairy cows resulted in an increase in Zn in milk but at a decreasing percent of Zn fed [115]. When 2000 ppm Zn was fed to dairy cows, milk yield and feed intake were decreased, milk and plasma Zn were higher, and plasma Cu was lower. Feeding 1000 ppm Zn had no negative effect [116].

An autosomal recessive trait, lethal Trait A46, occurs in Holstein and Shorthorn cattle breeds and results in a decreased ability to absorb Zn. Calves with this inherited trait have an acceptable number of functional lymphocytes at birth, but as the calves become Zn-deficient, lymphocyte activity is altered [117].

#### Swine

The overt signs and symptoms of Zn deficiency in swine are not seen when typical swine diets that meet the [66] recommendations are fed. Hence, many of the estimated Zn requirements did not result from a production setting research or today’s genetics.

Hoekstra et al. [118] reported that when 100 ppm Zn was added to a high Ca diet (1.6%) for bred gilts, the number of live pigs born was increased and Zn was increased in the serum, liver, and bone of dams and in the liver of the offspring. A dietary deficiency of Zn in reproducing females in the last trimester of pregnancy resulted in increased gestational length and parturition with low viability of offspring with abnormal bone tissue [119]. However, when sows were fed a corn-soybean meal diet for two parities, pigs per litter (live or total), birth weight, number weaned per litter, and their weaning weight did not differ from the performance of sows fed an additional 50 or 500 ppm Zn. However, there were more abnormal pigs born per litter if sows were not supplemented with Zn [120]. They reported fewer abnormal pigs per litter and almost an additional pig per litter when sows were supplemented with 500 ppm compared to 50 ppm Zn. When 700 ppm Zn sulfate was fed from day 80 of gestation until farrowing, stillbirth rate was decreased during prolonged farrowing [121].

Boars were reported to have higher Zn requirements than gilts or barrows [122] because of the role of Zn in male reproductive tissues and spermatogenesis.

As early as 1967, Ullrey et al. [123] reported that serum Zn concentrations ranged from 54 to 141 μg/100 ml. Hill et al. [124] noted that pigs at birth from sows not supplemented with Zn had 52 ppm hepatic Zn, but offspring from sows fed 5000 ppm Zn had 1300 ppm Zn regardless of parity (1 vs. 2). At 21 days of age, hepatic Zn increased in the offspring of sows fed 0, 50, or 500 ppm Zn compared to the decrease observed when dams were fed 5000 ppm Zn. Of importance is the Cu deficiency in the offspring of sows fed 5000 ppm Zn [67].

Several researchers reported that Zn oxide that is poorly absorbed fed at pharmacological concentrations stimulated growth, and they hypothesized that *Escherichia coli* scours were controlled. Smith et al. [125] showed that pigs weaned at about 15 days of age had improved average daily gain(ADG) with 3000 ppm Zn from Zn oxide but not when 250 ppm Cu was added to their diets as Cu sulfate. The North Central Regional Swine committee [126] also found a response to Cu and to Zn but not an additive growth response from the combination. Carlson et al. [127] reported that early weaned (12 days) or traditionally weaned pigs (25 days) needed 3000 ppm Zn as oxide for a minimum of 2 weeks immediately after weaning to get the increased performance. Additionally, her work showed that MT was increased in the liver leading to the likely role in metabolic management of Zn including protection against toxicity.

Rincker et al. [99] showed that when nursery pigs were fed 2000 ppm Zn either as Zn oxide or Zn methionine, fecal excretion did not increase until organs were “loaded” with Zn at approximately 14 days of supplementation. Similar Zn loading and excretion of Zn were observed by Martinez et al. [128] where duodenal MT was stimulated by 1000 and 4000 ppm Zn from oxide compared to the basal diet. Hence, pharmacological Zn can be fed for 2 weeks without potentially insulting the environment yet gaining increased performance.

When five sources of organic Zn, fed at 500 ppm, were compared to nursery pigs fed 500 and 2000 ppm Zn from Zn oxide in a regional study [129], the average daily feed intake (ADFI) and ADG were greater with 2000 ppm Zn from oxide than pigs fed any of the 500 ppm treatments. When various combinations and sources of Cu and Zn were fed, 3000 ppm Zn from oxide followed by 135 ppm Cu resulted in performance similar to feeding both for 42 days [130]. Meeting the suggested NRC weanling pig Zn requirements (1998) with or without additional Zn from various sources, van Heugten et al. [131] reported that immune response was adequate with all Zn sources fed. In nursery diets with traditional feedstuffs, Martin et al. [132] reported that diets must be supplemented with Fe, Se, and Zn to meet the pig’s requirements. Pigs weaned at 18 days of age were fed from 0 to 100 ppm Zn as either organic or inorganic or 50 ppm with equal contributions of both sources. Measuring MT throughout the GI tract and various antioxidant enzymes, the authors concluded that the newly weaned pigs managed Zn from sulfate and organic sources differently and that 75 ppm of organic Zn is necessary for today’s fast growing pigs with a very high lean tissue composition [133].

Differential expression in the livers of pigs fed pharmacological Zn of GLO1, PRDX4, and ACY1 mRNA was higher in pigs fed 1000 or 2000 ppm Zn vs.150 ppm [134]. Perhaps, this is an indication of how cell function and health are managed with pharmacological Zn feeding.

Feeding high levels of dietary zinc past a week may not improve piglet performance possibly caused by the observation of intestinal mRNA levels of zinc transporters decreasing (ZIP4) as well as increasing (ZnT1), which impaired zinc homoeostasis [135]. This theory is supported by the research of Pieper et al., [136] who reported digestive enzymes (alpha-amylase, lipase, trypsin, and chymotrypsin) and antioxidative capacity (TEAC or trolox equivalent antioxidative capacity) in the pancreas of pigs increased when pigs were fed high dietary zinc (2425 mg/kg).

Gowanlock et al. [137] showed that grow-finish pigs can meet their needs for Cu, Fe, and Mn from corn-soybean meal diets at this phase of production. However, Zn must be supplemented. They also found that hepatic and duodenal MT protein increased as the dietary microminerals increased in the diet, but jejunal MT did not increase. Their work provided changes in several antioxidant enzymes. Metallothionein and Zn transporters are expressed in mucosal duodenal cells when pigs were fed 0, 25, 50 ppm, and 100% of NRC, but no treatment differences were observed in this small study [138]. Metallothionein protein in duodenum and jejunum was not influenced when 0 to 100% of NRC trace element recommendations were fed, but hepatic MT was greatest when 25 ppm and 50% NRC were fed. Gilts had significantly less duodenal and jejunal MT than barrows [139]. This difference is concentration may be due to the role in reproduction of the gilt.

Whyte and Prater [140] noted that engineered Zn finger nucleases are used in genetically modifying pigs for agricultural and biomedical research.

#### Poultry

In 1958, O’Dell and colleagues [141] reported the signs of Zn deficiency in chickens developed in 4 to 6 weeks of being fed 15 ppm in galvanized batteries. When the access to Zn in cage coating was removed, more severe deficiency symptoms developed. The reported symptoms included reduced growth, shortening and thickening of long bones, poor feather development, and rapid and labored breathing. The phytic acid content of soybeans makes Zn less available to non-ruminants and hence today the enzyme, phytase, is used in diets.

Oh et al. [142] reported that chicks fed high concentrations of Zn had MT in the liver, kidney, pancreas, and intestinal mucosa. When birds fed high Zn diets were switched to low Zn diets, the Zn in MT was no longer present indicating the role of MT in Zn homeostasis in the chick.

Wang et al. [143] found that Zn deficiency in chicks has a direct effect on proliferation, differentiation, and apoptosis on growth plate chondrocytes; hence the shortened bones observed by the O’Dell and Savage laboratories. Alkaline phosphatase activity is reduced during Zn deficiency in the bones of turkey poults [144]. Bettger et al. [145] suggested extracellular Zn has an effect on water metabolism when studying Zn deficiency.

Large quantities of Zn (1000 ppm) from feed or gavage doses were compared relative to concentration in tissues of chicks. The Zn accumulations were greatest in bone, pancreas, kidney, and liver [146]. It should be noted that high concentrations of Zn (3000 to 4000 ppm) can affect the adenohypophyseal gonadotropic hormone, adrenal glands, and exocrine and endocrine portions of the pancreas. After 2 weeks of adequate Zn, functions of these organs returned to normal [147]. Lü and Combs [148] found that the acinar portion of the pancreas suffered structural changes with Zn toxicity. Kincaid et al. [149] noted that up to 1200 ppm dietary Zn was managed homeostatically by the chick, but Zn metabolism was altered with 2400 ppm Zn.

Hens needed 65 ppm Zn and 4.0% Ca to produce normal chicks. Without Zn supplementation, if Zn-deficient chicks hatched, they would be weak and unable to stand, eat, and drink [150]. The Zn status of the breeder hens influences hatchability, and the use of Zn during incubation influences the development and functionality of the immune system [151–153]. The production of hens through four layer years was not affected by the addition of 0, 10, 20, or 40 ppm to a basal diet of 28 to 34 ppm. Hence, suggesting that 28 ppm Zn provided by a corn-soybean diet may be adequate for hens of this genotype [154].

When mature laying hens were fed 20,000 ppm Zn for 4 days, ovary and oviduct weights were reduced by 10 days. By 18 days, liver and kidney Zn concentrations returned to pre-treatment concentrations, but the pancreas maintained its high concentration [155]. This is a means of forced rest [156]. However, Dewar et al. [157] reported that after 4 days of 10,000 ppm dietary Zn, lesions were found in the gizzard and pancreas of hens. These disappeared when the excessive Zn was removed from the diet for 28 days. Eggs from hens fed 10,000 or 20,000 ppm Zn had lower concentrations of K, Cu, and Zn than controls fed 20 ppm Zn [158].

#### Horses

Because most horses are utilized as a recreational/companion animal in the USA and not for meat production and ranch or work, there is limited research done with Zn. The NRC recommendation of 9 ppm Zn does not appear to cause osteochondrosis in the short-term studies that have been completed. However, when excessive Zn and Cd polluted the soil near a smelter, horses were determined to have osteochondrosis likely due to abnormal Cu metabolism [159] as seen in sows fed 5000 ppm Zn for two parities [67].

Ott and Asquith [160] reported that trace mineral supplementation (Fe, Zn, Mn, Cu) is likely necessary in many diets if their diet is below the recommendations of the NRC (40 ppm). In a small sample size, milk from mares collected weekly (week 1 to 8), the Zn and Cu concentrations decreased weekly, but concentrations in the initial colostrum were not measured [161].

### Interrelationship of Zn with Other Minerals

The effect of high and/or pharmacological Zn on Cu metabolism is seen in all species. In sheep, it is useful in reducing marginal Cu toxicity. As noted earlier, in swine, it results in bone fragility when fed for several months, and in poultry [162] when Zn was fed in excess, lysis of RBC was increased and the antioxidant enzyme Cu Zn SOD activity was depressed. The addition of Cu reversed these metabolic changes.

Chicks fed a low Zn diet with soybean protein developed Zn deficiency signs such as skin and joint pathology. Excessive dietary vitamin E decreased peroxidative products and decreased the severity that was observed [163, 164].

Rama and Planas [165] reported that oral pharmacological Zn interfered with Cu and Fe metabolism in the chick that could be overcome if IM injections of Cu and Fe were given. Chicks fed excess Zn retained less Fe in tibia and Cu in the liver and pancreas, demonstrating the three-way interaction of Cu/Zn/Fe seen in other species [162, 166]. Blalock and Hill [167] found Zn to be more toxic in chicks that were Fe-deficient. In chicks supplemented with Fe, more Zn was bound on MT than in Fe-deficient chicks.

As observed in the Japanese quail, the accumulation of Cd in the liver and kidney is reduced by supplementing diets with Zn, Cu, and Mn [168].
